# Quality use of medicines in patients with chronic kidney disease

**DOI:** 10.1186/s12882-020-01862-1

**Published:** 2020-06-05

**Authors:** Ronald L. Castelino, Timothy Saunder, Alex Kitsos, Gregory M. Peterson, Matthew Jose, Barbara Wimmer, Masuma Khanam, Woldesellassie Bezabhe, Jim Stankovich, Jan Radford

**Affiliations:** 1 Pharmacology and Clinical Pharmacy, University of Sydney, Sydney School of Nursing, Camperdown, Sydney, 2000 Australia; 2 Blacktown Hospital, Blacktown, New South Wales Australia; 3 School of Medicine, University of Tasmania, Private Bag 34, Hobart, TAS 7001 Australia; 4 Faculty of Health Science, University of Tasmania, Churchill Avenue, Sandy Bay, TAS 7001 Australia; 5 Faculty of Medicine, University of Tasmania, Private Bag 96, Hobart, TAS 7001 Australia; 6 Division of Medicine, Royal Hobart Hospital, Private Bag 96, Hobart, TAS 7001 Australia; 7 School of Medicine, University of Tasmania, Private Bag 26, Hobart, TAS 7001 Australia; 8 School of Health Sciences, University of Tasmania, Churchill Avenue, Sandy Bay, TAS 7005 Australia; 9 School of Medicine, University of Tas, Private Bag 26, Hobart, TAS 7001 Australia; 10 Department of Neuroscience, Monash University, 99 Commercial Rd, Melbourne, VIC 3004 Australia; 11 Launceston Clinical School, School of Medicine, Locked Bag 1377, Launceston, Tas 7250 Australia

**Keywords:** Inappropriate prescribing, Drug dosing, Kidney disease

## Abstract

**Background:**

Chronic kidney disease (CKD) affects drug elimination and patients with CKD require appropriate adjustment of renally cleared medications to ensure safe and effective pharmacotherapy. The main objective of this study was to determine the extent of potentially inappropriate prescribing (PIP; defined as the use of a contraindicated medication or inappropriately high dose according to the kidney function) of renally-cleared medications commonly prescribed in Australian primary care, based on two measures of kidney function. A secondary aim was to assess agreement between the two measures.

**Methods:**

Retrospective analysis of routinely collected de-identified Australian general practice patient data (NPS MedicineWise MedicineInsight from January 1, 2013, to June 1, 2016; collected from 329 general practices). All adults (aged ≥18 years) with CKD presenting to general practices across Australia were included in the analysis. Patients were considered to have CKD if they had two or more estimated glomerular filtration rate (eGFR) recorded values < 60 mL/min/1.73m^2^, and/or two urinary albumin/creatinine ratios ≥3.5 mg/mmol in females (≥2.5 mg/mmol in males) at least 90 days apart. PIP was assessed for 49 commonly prescribed medications using the Cockcroft-Gault (CG) equation/eGFR as per the instructions in the Australian Medicines Handbook.

**Results:**

A total of 48,731 patients met the Kidney Health Australia (KHA) definition for CKD and had prescriptions recorded within 90 days of measuring serum creatinine (SCr)/estimated glomerular filtration rate (eGFR). Overall, 28,729 patients were prescribed one or more of the 49 medications of interest. Approximately 35% (*n* = 9926) of these patients had at least one PIP based on either the Cockcroft-Gault (CG) equation or eGFR (CKD-EPI; CKD-Epidemiology Collaboration Equation). There was good agreement between CG and eGFR while determining the appropriateness of medications, with approximately 97% of the medications classified as appropriate by eGFR also being considered appropriate by the CG equation.

**Conclusion:**

This study highlights that PIP commonly occurs in primary care patients with CKD and the need for further research to understand why and how this can be minimised. The findings also show that the eGFR provides clinicians a potential alternative to the CG formula when estimating kidney function to guide drug appropriateness and dosing.

## Background

The prevalence of chronic kidney disease (CKD) is rapidly increasing worldwide, alongside its complications [1–3]. Most people with CKD are prescribed a multitude of drugs to treat the underlying cause of kidney disease, or its numerous complications and comorbidities [2, 3]. The reduction in kidney function requires adaptation of treatment regimens as the pharmacokinetics and pharmacodynamics of many drugs are altered. Dosing errors are common in CKD and can cause adverse effects and poor outcomes [2].

Previous studies have reported potentially inappropriate prescribing (PIP) of medications in CKD (defined as the use of a contraindicated medication or inappropriately high dose according to the kidney function) ranging from 13 to 80%, with most of the medications being initiated in the community setting [2, 4]. In Australia, the prevalence of PIP in patients with renal impairment in primary care has received less attention. One study reported that 32% of patients with CKD were receiving PIP at the time of admission to hospital [2].

The automated reporting of estimated glomerular filtration rate (eGFR) calculated from the modification of diet in renal disease (MDRD) or CKD epidemiology collaboration equation (CKD-EPI) is now routinely provided by laboratories whenever a serum creatinine (SCr) value is measured, as a tool to enhance the identification and classification of CKD [5]. Most of the drug dosing studies to date, however, have used the Cockcroft-Gault (CG) equation to estimate kidney function as creatinine clearance (CrCl), which includes a weight component [6]. While the CG equation is still the preferred equation for drug dosage adjustment by most drug information sources, it is cumbersome to calculate in the busy context of a general practice consultation.

Given this background, the main objective of this study was to determine the extent of PIP of renally-cleared medications commonly prescribed in Australian primary care, based on two separate estimates of kidney function (CG and CKD-EPI).

## Methods

This retrospective study included data collected by MedicineInsight, developed and managed by NPS MedicineWise. MedicineInsight is a large-scale national data program in Australia to extract and collate longitudinal, whole-of-practice data from the clinical information systems of consenting general practices. De-identified patient data collected include demographics, encounters (not including progress notes), diagnoses, prescriptions and pathology tests. As of July 2017, MedicineInsight had recruited 650 general practices with data pertaining to 3.6 million patients [3, 7, 8]. The Institutional Human Research Ethics Committee approved the study (H0015651). The study is reported as per the reporting of studies conducted using observational routinely collected health data statement for pharmacoepidemiology (RECORD-PE) guideline [9].

We used MedicineInsight data from January 1, 2013 to June 1, 2016, collected from 329 general practices distributed across Australia. Patients were initially included if at the time of data extract they were aged at least 18 years, met the Royal Australian College of General Practice (RACGP) definition of an “active” patient (attended at least three times at the same general practice within a two-year period), and could be diagnosed with CKD based on their laboratory pathology results (having two or more eGFR values less than 60 mL/min/1.73m^2^, and/or two urinary albumin/creatinine ratios (ACR) ≥ 3.5 mg/mmol in females or ≥ 2.5 mg/mmol in males, at least 90 days apart) [1].

A total of 60,433 patients out of 1.48 million (approximately 4%) in the dataset met the Kidney Health Australia (KHA) guidelines for the diagnosis of CKD [1]. For assessing the quality use of medicines (QUM) in kidney disease we included a total of 49 medications, which were part of a national campaign to improve prescribing in the elderly in 2012 [10]. The list was adapted to include some medications that were commonly associated with PIP in other research [2]. Patients’ medications were included if they were prescribed within 90 days following the pathology test. PIP was defined as prescribing of a medication that is contraindicated or at an inappropriately high dose according to the patient’s kidney function. Appropriateness was assessed using the Australian Medicines Handbook (AMH) [11]. Only medications that had clear recommendations were included in the analysis. Medications with ambiguous dosage adjustment recommendations were excluded from the analysis e.g. *Ramipril- “In patients with renal impairment, elderly or taking a diuretic, initially 2.5mg once daily”* [11]. Additionally, medications that were available in combination were assessed for PIP separately. For example; metformin and sitagliptin were assessed for PIP and reported separately. Examples of drugs and recommendations extracted from the AMH are presented in Additional file 1. The complete list of medications and recommendations is available on request.

### Statistical analysis

Descriptive statistics were used to describe the data, with mean ± standard deviation (SD) and percentages/proportions. PIP of medications, i.e. both potentially contraindicated and inappropriately dosed medications, were compared for appropriateness between the CKD-EPI and CG equations (patients with weight and height documented). The level of agreement in evaluating appropriateness between the equations was calculated by Cohen’s kappa and Gwet first-order agreement coefficient (AC1) [12]. For Cohen’s Kappa, a value of < 0.6 was defined as poor agreement, 0.6–0.8 as moderate agreement, 0.8 to < 0.9 as good agreement and ≥ 0.9 as excellent agreement. Gwet AC1 provides an alternative in circumstances where Kappa is low despite a high level of agreement and has been shown to provide a more stable inter-rater reliability coefficient than Cohen’s kappa [12]. All data cleaning and manipulation, and statistical analyses were completed using the statistical and graphical computing language of R [13]. A *p* value of < 0.05 was considered statistically significant.

## Results

A total of 48,731 patients met the KHA definition for CKD and had prescriptions recorded within 90 days of measuring kidney function. Overall, 28,729 patients were prescribed one or more of the 49 medications of interest (Table 1). Over 90% of these patients (*n* = 25,975) were ≥ 65 years of age and 55.7% (*n* = 15,993) were female. The mean (SD) number of medications and medical conditions were 8.4 (3.8) and 2.2 (1.0), respectively. Over 80% (*n* = 23,998) of the patients had hypertension, while over 40% had diabetes and/cardiovascular disease (*n* = 13,292 and 13,611, respectively). Among the 28,729 patients, almost all 98.5% (*n* = 28,315) were in Stage 3, 4 and 5 CKD. The most common drug class prescribed across the study sample were agents pertaining to the renin-angiotensin system (RAS).

**Table 1:** Characteristics of the study sample (*n* = 28,729)

Demographics	Number (%)
Age (years)	
20–29	6 (0.02)
30–39	61 (0.21)
40–49	257 (0.89)
50–59	1039 (3.62)
60–69	4234 (14.74)
70–79	10,219 (35.57)
80–89	10,434 (36.32)
90+	2479 (8.63)
Male Gender	12,736 (44.3)
CKD Risk Factors	
Hypertension	23,998 (83.2)
CVD	13,611 (47.4)
Diabetes	13,292 (46.3)
Number of medications [Mean (SD)]	8.42 (3.75)
Top 5 medications categorised according to the ATC groups	
Agents acting on the renin-angiotensin system	20,391 (71.0)
Analgesics	16,084 (56.0)
Drugs for acid-related disorders	15,861 (55.2)
Antithrombotic drugs	11,691 (40.7)
Diuretics	10,965 (38.2)
Stages of CKD	
Stage 1 ≥ 90 mL/min/1.73m^2^	27 (0.1)
Stage 2 60–89 mL/min/1.73m^2^	387 (6.8)
Stage 3a 45–59 mL/min/1.73m^2^	18,553 (64.6)
Stage 3b 30–44 mL/min/1.73m^2^	7539 (26.2)
Stage 4 15–29 mL/min/1.73m^2^	1955 (6.8)
Stage 5 < 15 mL/min/1.73m^2^	268 (0.9)

*CVD* Cardiovascular disease, *ATC* Anatomical Therapeutic Chemical classification system

### Potentially inappropriate prescribing

**Fig. 1:**
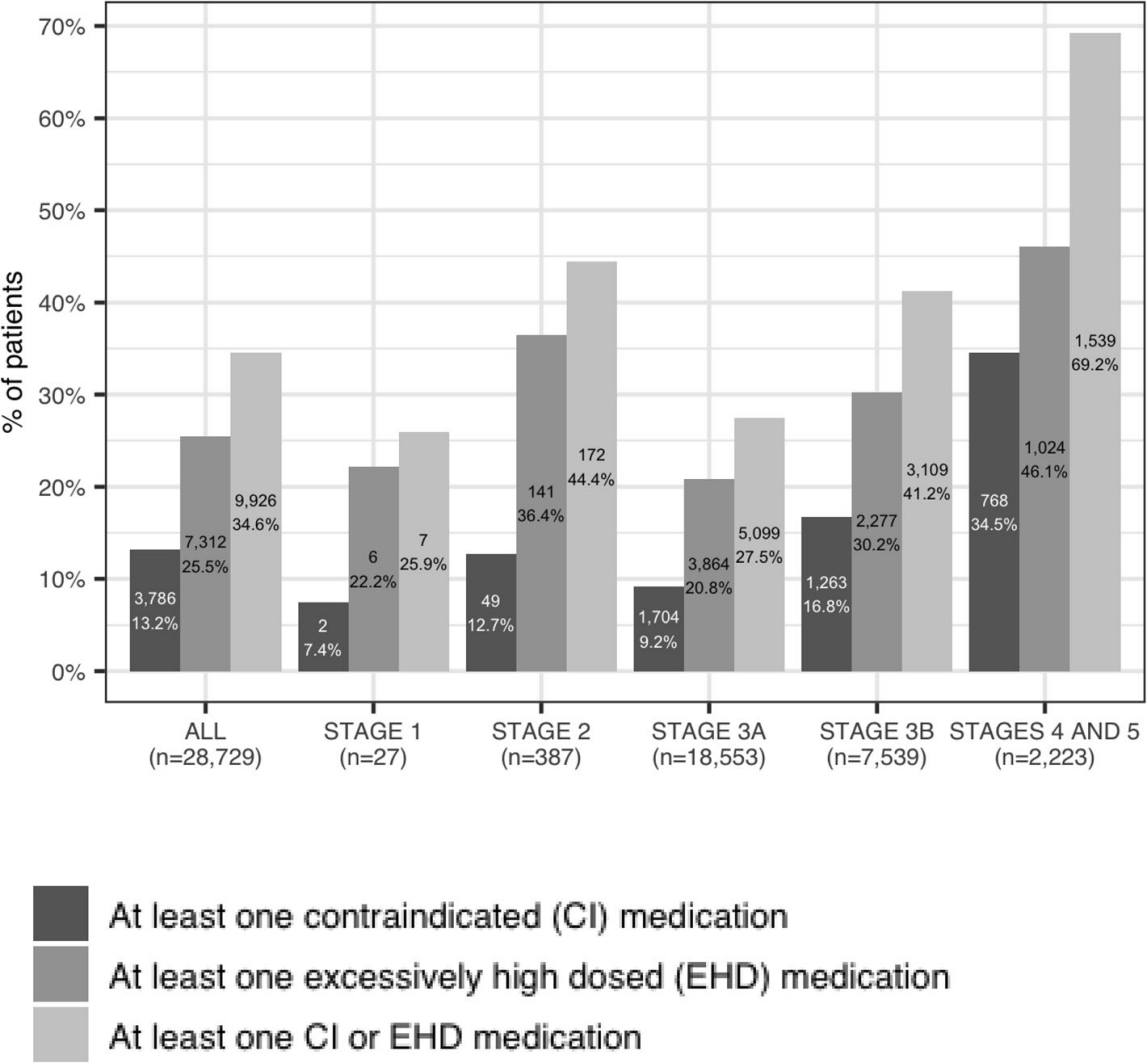
Potential inappropriate prescribing as per CKD stage

A breakdown of PIP across the different stages of CKD is provided in Fig. 1. Overall, 35% (*n* = 9926) patients had at least one PIP based on either CG CrCl or eGFR. Approximately 6.5% (*n* = 1866) and 1.8% (*n* = 505) were prescribed 2 and 3 or more potentially inappropriate medications (PIMs), respectively (Table 2). A total of 1171 patients (4.1%) were prescribed both a contraindicated medication and a medication at an inappropriate dose as per their kidney function.

**Table 2:** Potentially inappropriate medications (PIMs) in the study sample

Characteristic	Number (%)
PIMs	
0	18,803 (65.4)
1	7555 (26.3)
2	1866 (6.5)
≥ 3	505 (1.8)
Patients with potentially inappropriate dose	7312 (25.5)
0	21,417 (74.5)
1	5983 (20.8)
2	1143 (4.0)
≥ 3	186 (0.6)
Patients with potentially contraindicated medication	3786 (13.2)
0	24,943 (86.8)
1	3494 (12.2)
2	265 (0.9)
≥ 3	27 (0.1)
Patients with both potentially inappropriate dose and contraindicated medication	1171 (4.1)

Analgesics, prescribed across 7.2% patients (*n* = 2082), were the most frequent inappropriate class of medication [*n* = 1536 (73.8% of patients prescribed the drugs)] followed by medications for diabetes [prescribed *n* = 8730 (30.4%); PIP *n* = 3384 (44.1%)] and beta-blockers [prescribed *n* = 4832 (16.8%); PIP *n* = 1849 (38.2%)]. Vildagliptin [prescribed *n* = 355 (1.2%); PIP *n* = 274 (77.2%)] followed by fenofibrate [prescribed *n* = 1939 (6.7%); PIP *n* = 1346 (69.4%)] were the most commonly prescribed medications at an inappropriate dose, whilst dapagliflozin [prescribed *n* = 296 (1%); PIP *n* = 245 (82.8%)] followed by alendronate [prescribed *n* = 791(2.8%); PIP *n* = 251 (31.7%)] had the highest rates of use when contraindicated either by CG CrCl or eGFR (Table 3)**.** Codeine, glibenclamide and glimepiride were associated with PIP in all patients prescribed these drugs, as the AMH recommends their avoidance in renal impairment, and these were excluded from the final analysis.

**Table 3:** Medications with potentially inappropriate prescribing

Medications with potentially inappropriate dose	Patients prescribed N (%)	Patients with inappropriately high dose drug N (%)	Medications potentially contraindicated	Patients prescribed N (%)	Patients with potentially contraindicated medication N (%)
**Antidiabetic Medications**			**Antidiabetic Medications**		
Vildagliptin	355 (1.2)	274 (77.2)	Dapagliflozin	296 (1.0)	245 (82.8)
Saxagliptin	205 (0.7)	117 (57.1)	Acarbose	81 (0.3)	12 (14.8)
Sitagliptin	1534 (5.3)	742 (48.4)	Exenatide	313 (1.1)	18 (5.8)
Metformin	8046 (28.0)	2885 (35.9)	Metformin	8046 (28.0)	37 (0.5)
Alogliptin	17 (0.1)	5 (29.4)	**Anticoagulant Medications**		
**Lipid Lowering Medications**			Rivaroxaban	1230 (4.3)	99 (8.0)
Fenofibrate	1939 (6.7)	1346 (69.4)	Dabigatran	492 (1.7)	34 (6.9)
Rosuvastatin	8529 (29.7)	525 (6.2)	Apixaban	938 (3.3)	40 (4.3)
**Antihypertensive Medications**			**Cardiovascular Medications**		
Atenolol	4832 (16.8)	1849 (38.3)	Moxonidine	1675 (5.8)	447 (26.7)
Moxonidine	1675 (5.8)	43 (2.6)	Spironolactone	3050 (10.6)	665 (21.8)
**Antiarrhythmics**			**Bisphosphonates**		
Digoxin	2567 (8.9)	24 (0.9)	Alendronate	791 (2.8)	251 (31.7)
**Anticoagulant Medications**			Risedronate	619 (2.2)	118 (19.1)
Rivaroxaban	1230 (4.3)	237 (19.3)	**Others**		
Dabigatran	492 (1.7)	39 (7.9)	Teriparatide	7 (0.0)	2 (28.6)
**Bisphosphonates**			Strontium	205 (0.7)	43 (21.0)
Clodronate	10 (0.03)	5 (50)	Dextropropoxyphene	105 (0.4)	33 (31.4)
Zoledronate	24 (0.1)	1 (4.2)	Pramipexole	523 (1.8)	100 (19.1)
**Antihistamines**			Probenecid	115 (0.4)	1 (0.9)
Nizatidine	283 (1.0)	170 (60.1)			
Cimetidine	15 (0.1)	3 (20.0)			
Cetirizine	261 (0.9)	50 (19.2)			
**Psychotropic Medications**					
Paliperidone	6 (0.02)	4 (66.7)			
Desvenlafaxine	572 (2.0)	70 (12.2)			
Duloxetine	846 (2.9)	87 (10.3)			
Venlafaxine	1050 (3.7)	47 (4.5)			
Varenicline	150 (0.5)	6 (4.0)			
**Neurological Medications**					
Tramadol	571 (2)	67 (11.7)			
Gabapentin	341 (1.2)	30 (8.8)			
Levetiracetam	179 (0.6)	9 (5)			
Pregabalin	4642 (16.2)	190 (4.1)			
Pramipexole	523 (1.8)	3 (0.6)			
***Others***					
Tolterodine	20 (0.1)	2 (10.0)			
Solifenacin	458 (1.6)	21 (4.6)			

*Codeine, Glibenclamide and Glimepiride were prescribed inappropriately in all patients and is not presented in the table; Combination medications were assessed for PIP separately*

### Agreement between eGFR (CKD-EPI) and CrCl (CG equation)

**Fig. 2:**
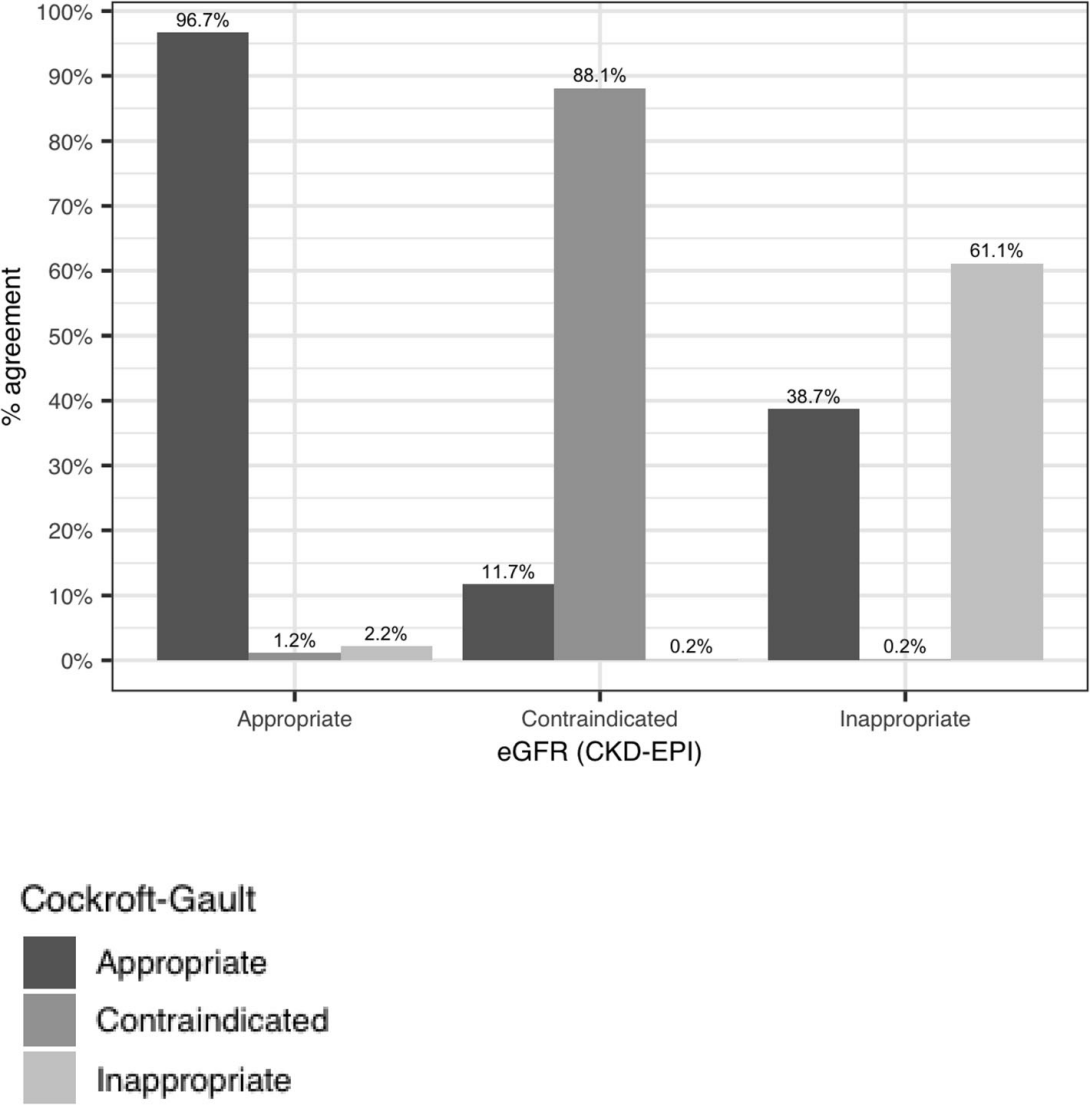
Agreement of equations in determining PIP

Overall, there was good agreement between CG CrCl and eGFR (CKD-EPI) when determining the appropriateness of medications. Approximately 97% of medications that were classified as appropriate by eGFR were considered to be appropriate by the CG equation, while 88.1 and 61.1% of the medications considered to be contraindicated and inappropriately dosed by eGFR, were considered to be contraindicated and inappropriately dosed by the CG equation, respectively **(**Fig. 2). The top 3 medications in disagreement between the two equations are presented in Table 4. A total of 40 medications from the 49 of interest, that had been rated PIP at least once, were tested for inter-rater agreement. Twenty-seven medications scored a Cohen’s kappa value < 0.6 whereas Gwet’s AC1 scores were between 0.75–1.0.

**Table 4:** Top 3 medications considered in disagreement between CG CrCl and eGFR (CKD-EPI)

CKD-EPI eGFR	CG Equation	Medication	N (%)
Appropriate	Contraindicated	Spironolactone	229 (27.8)
		Alendronate	186 (22.5)
		Risedronate	105 (12.7)
	Inappropriate dose	Atenolol	501 (32.3)
		Metformin	272 (17.5)
		Sitagliptin	169 (10.9)
**Contraindicated**	Appropriate	Moxonidine	270 (30.5)
		Spironolactone	241 (27.3)
		Dapagliflozin	177 (20)
	Inappropriate dose	Moxonidine	7 (46.7)
		Metformin	4 (26.7)
		Rivaroxaban	4 (26.7)
**Inappropriate dose**	Appropriate	Metformin	1812 (35.5)
		Fenofibrate	915 (17.9)
		Atenolol	675 (13.2)
	Contraindicated	Rivaroxaban	13 (52.0)
		Metformin	12 (48.0)

## Discussion

The results demonstrate that Australian general practice patients with CKD are frequently prescribed PIP. Approximately 35% of the patients were prescribed at least one PIP. The findings have corroborated the high rate of potentially contraindicated medications and inappropriate dosing found in international studies, including a systematic review that reported rates of PIP between 13 to 80% [2, 4]. These results highlight the need for further research to determine the reasons, leading to interventions to optimise prescribing in patients with CKD in general practice.

The extent of PIP in our study and the previous literature highlights the complexity of prescribing in CKD and could be due to the contribution of several factors. Firstly, in Australia there is a lack of up-to-date accessible guidelines on drug dose adjustments in patients with impaired kidney function. Another important aspect to consider is the inconsistencies with dosage adjustment recommendations between different drug information sources, as well as the product information of different brands of the same drug [14, 15]. It is important to note that most drug information sources provide dosage adjustment based on the CG equation rather than eGFR formulae (MDRD/CKD-EPI), whilst eGFR (especially CKD-EPI) based formulae have been found to be the most accurate indicator of kidney function [16]. Secondly, many of the recommendations based on the CG equation are questionable due to the variability in creatinine assays at the time and were prior to SCr being isotope dilution mass spectrometry (IDMS) standardised. CG-estimated GFR results are 5–10% higher using the standardised SCr measurements compared to the non-standardised SCr and relying on the CG equation could lead to unintended consequences, including insufficient dose adjustments for kidney function [17, 18]. However, the clinical significance of this theoretical issue is unknown and needs to be evaluated further. Hence, current recommendations include using either CG equation due to considerable experience with the formula even if it is based on creatinine assays not in use. It is also appropriate to use eGFR formula (CKD-EPI) for most drugs without body surface area (BSA) adjustment [1, 16].

The nature of the electronic health record (eHR) may also be a potential factor for PIP. The current eHR may not alert prescribers to appropriately adjust dosages of medications cleared by the kidney unless the patient has CKD recorded as a diagnosis. Only 20% of the patients included had a formal diagnosis of CKD documented whilst all patients had laboratory evidence of CKD and this lack of coding of CKD as a condition may have had a role in the PIP findings [7, 8]. Whilst the overall prevalence of CKD in our study was comparable to the previous Australian Bureau of Statistics (ABS) results, the prevalence is lower than previous International literature. The potential reasons for the lower prevalence have been described in detail elsewhere [3]. It is important to note that in many older people with stable eGFR values from 45 to 59 mL/min/1.73 m^2^ debate on the definition and staging of CKD exists as to whether CKD may be over diagnosed. However, these values are a sign of impaired kidney function that could affect the clearance of drugs. Hence, it is essential to consider renal function when prescribing renally-cleared medications as the PIP may lead to adverse outcomes.

The agreement between the two equations (CG and CKD-EPI) was excellent, with 97% of the medications rated as appropriate by eGFR being also rated as appropriate by the CG equation. Previous studies comparing drug doses derived from eGFR equations and CG have commonly reported discordance rates between 10 and 40% [19, 20]. These results highlight that for most patients with renal impairment the same recommendation between eGFR or CG estimate can be used, with any difference in the kidney function estimate unlikely to lead to an overdose [21] However, it is important for some drugs, and the potential clinical significance of disagreement between eGFR and CG-based dosing regimens should be minimised by using sound clinical judgment [22]. For example, our study showed that rivaroxaban was one of the medications associated with disagreement between the equations. A recent study by Szummer et al. comparing eGFR (CKD-EPI, MDRD) and the CG equation suggested that when prescribing one of the novel oral anticoagulants, the CG equation should be considered, as it provides a more conservative approach for avoiding drug exposure and reducing the risk of bleeding [23]. A similar approach is recommended by the National Kidney Disease Education Program (NKDEP) in the USA and by KHA, where eGFR can be used for dosage adjustment for most drugs (without BSA adjustment) except for drugs with a narrow therapeutic index (e.g. anticoagulants), where conservative kidney function estimates and corresponding doses are recommended, particularly if therapeutic drug monitoring is not readily available [16].

Most of the medications with PIP were consistent with previous studies [2]. Antidiabetic agents and cardiovascular drugs were commonly associated with PIP, which is not surprising given that both diabetes and CVD often co-exist with CKD. Almost half of our patients were being treated for diabetes and CVD. A large proportion of oral antidiabetic agents are excreted by the kidney [11]. It is likely that concomitant chronic diseases complicate and confound the treatment of each other. These medications are adjusted frequently in response to cardiac, renal or electrolyte disturbances, and are at a higher risk of being potentially inappropriate.

It is important to note that for some medications commonly prescribed in the study (for example, atenolol) clinical markers, such as heart rate and blood pressure, are often more important than kidney function in guiding dosage adjustment. Additionally, some medications do not have adequate published data in patients with renal impairment and may have been conservatively recommended as to be avoided rather than having an increased potential for toxic effects [11].

Our study also showed that PIP was common across all stages of CKD. Although stages 1, 2 and 3a CKD seldom impact on the prescribing of renally cleared medications, several common medications such as metformin, gliptins and SGLT2 inhibitors do have specific recommendations in patients with early stages of CKD. Previous studies in patients with advanced CKD have shown contrasting results. Some studies have reported that in patients with advanced CKD the likelihood of PIP is less due to more vigilant monitoring and nephrology consultations, while other studies have shown an increased number of problems [24–26]. Further research to understand the phenomenon of PIP in general practice is needed, possibly leading to the design of interventions to optimise medication prescribing in patients with CKD. Optimal use of current eHR systems to flag renally-impaired patients so that PIP is identified is likely to be part of an intervention.

The major strength of the current study is that it assessed prescriptions in primary care in a large cohort of patients. Previous Australian studies of inappropriate drug prescriptions in CKD have focussed on older people in the community and aged care settings, or when patients were admitted to the hospital [4]. However, the study does have some limitations. Prescribers may not always have had pathology results available at the time of prescribing. However, we evaluated the extent of PIP for medications prescribed within 90 days of the availability of a kidney function assessment. We did not examine any adverse clinical outcomes associated with PIP. Additionally, prescribing may be appropriate at times with benefits outweighing the additional risks or if no safer alternatives are available.

## Conclusion

Our study highlights that PIP is common in Australian general practice, underlining the complexity of prescribing for patients with CKD. Understanding situations where prescribing is truly inappropriate would assist in the design of interventions to improve safe medication prescribing in patients with CKD. Our study also shows that although CrCl using the CG formula has been the most common method of estimating kidney function for drug dosing purposes for over 40 years, the widespread availability and extensive clinical use of eGFR now provides clinicians with a potential alternative. However, careful consideration of the risk-benefit ratio of individual drugs and doses within each patient is warranted.

## Supplementary information

**Additional file 1.** Examples of renally cleared medications and dosage recommendations from the AMH.

**Additional file 2.**

## Data Availability

Yes, if required after appropriate grant of permission from MedicineInsight.

## Abbreviations

AC1: Agreement coefficient
ACR: Albumin/creatinine ratio
AMH: Australian Medicines Handbook
BSA: Body surface area
CVD: Cardiovascular disease
CKD: Chronic kidney disease
CKD-EPI: CKD-Epidemiology Collaboration Eq.
CG: Cockcroft-Gault
CrCl: Creatinine clearance
eHR: electronic health record
eGFR: estimated glomerular filtration rate
IDMS: Isotope dilution mass spectrometry
KHA: Kidney Health Australia
MDRD: Modification of diet in renal disease
NKDEP: National Kidney Disease Education Program
PIP: Potentially inappropriate prescribing
QUM: Quality use of medicines
RACGP: Royal Australian College of General Practice
SCr: Serum creatinine
SGLT2: Sodium glucose cotransport inhibitors
