# Supplementary material for: Quality use of medicines in patients with chronic kidney disease
Source: BMC Nephrol. 2020 Jun 5;21:216. doi: 10.1186/s12882-020-01862-1 (PMC7275522; doi:10.1186/s12882-020-01862-1)
Supplement: Supplementary file 1 — Additional file 1. Examples of renally cleared medications and dosage recommendations from the AMH. [file 12882_2020_1862_MOESM1_ESM.docx]

| Medications | Recommendations |
| --- | --- |
| ALENDRONATE | Cr Cl Contraindicated if CrCl <35ml/min |
| DABIGATRAN | Cr Cl 30-50ml/min: 110mg TWICE daily maximum  Cr Cl <30ml/min: Contraindicated |
| SITAGLIPTIN | Cr Cl 30-50ml/min: 50mg once daily  Cr Cl <30ml/min: 25mg once daily |
| METFORMIN | Cr Cl 60-90ml/min: 2 grams maximum  Cr Cl 30-60ml/min: 1 gram maximum  Cr Cl 15-30ml/min: 500mg maximum  Cr Cl <15ml/min: Contraindicated |
| FENOFIBRATE | Cr Cl 20-60 mL/minute, oral 96 mg once daily  Cr Cl 10-20 mL/minute, oral 48 mg once daily |
| DIGOXIN | Cr Cl 30-60ml/min: 62.5–250mcg daily  Cr Cl 10-30ml/min: 62.5–125mcg daily  Cr Cl <10ml/min: 62.5 micrograms once daily or on alternate days |
| ROSUVASTATIN | Cr Cl <30ml/min, 10mg maximum |
| LEVETIRACETAM | Cr Cl 50-79ml/min: 500-1000mg twice daily  Cr Cl 30-49ml/min: 250-750mg twice daily  Cr Cl <30ml/min: 250-500mg twice daily  Dialysis: 500-1000mg once daily |
| PREGABALIN | Cr Cl 30-60ml/min: 300mg maximum  Cr Cl 15-30ml/min: 150mg maximum  Cr Cl <15 ml/min: 75mg maximum |
| DULOXETINE | Cr Cl <30ml/min: 30mg daily |

APPENDIX A: Examples of renally cleared medications and dosage recommendations from the AMH

CrCl: Creatinine Clearance; ** recent update of small dose is permitted below 30ml/min was not included;
